# Comprehensive analysis of the importance of PLAUR in the progression and immune microenvironment of renal clear cell carcinoma

**DOI:** 10.1371/journal.pone.0269595

**Published:** 2022-06-08

**Authors:** Zhiwei Wang, Kunxiong Wang, Xin Gao, Zhenxiang Liu, Zengshu Xing

**Affiliations:** 1 Department of Urology, Affiliated Haikou Hospital of Xiangya Medical College, Central South University, Haikou, Hainan, China; 2 Graduate School of Peking Union Medical College, Chinese Academy of Medical Sciences, Tsinghua University, Beijing, China; 3 Clinical Laboratory, The First People’s Hospital of Huaihua, Huaihua, Hunan, China; Roswell Park Cancer Institute, UNITED STATES

## Abstract

Clear cell renal cell carcinoma (ccRCC) is a common type of kidney cancer with a high mortality rate, and the discovery of new therapeutic markers is essential to improve patient survival. The plasminogen activator urokinase receptor (PLAUR) plays key roles in tissue remodeling and extracellular matrix degradation, which contribute to invasion and metastasis, a major feature of tumor malignancy. The role of PLAUR in ccRCC pathology has not been deeply studied. In this study, we collected the mRNA expression data of 33 tumor types, each derived from human patients obtained from TCGA database, and comprehensively analyzed the correlation between the expression of PLAUR in tumors and prognosis. Then, we studied the relationship between PLAUR expression in ccRCC and specific clinical features of ccRCC patients. In addition, we analyzed the function and mechanism of PLAUR in ccRCC. Our results showed that PLAUR was significantly overexpressed in ccRCC and that both PLAUR levels and PLAUR methylation levels significantly correlated with poor prognosis. Our results also suggest that PLAUR is involved in the progression of ccRCC. The results of functional and mechanistic analysis of PLAUR showed that PLAUR is involved in inflammatory and immune-related pathways in ccRCC; other data showed that PLAUR expression may affect the infiltration of multiple immune cell types in ccRCC and that PLAUR levels were significantly and positively correlated with the expression of immune checkpoints. In conclusion, our findings suggest that high PLAUR expression can promote the progression of ccRCC to poor prognosis, and thus PLAUR may serve as both a potential marker for predicting macrophage infiltration and immune microenvironment status and as an important immunotherapy target for ccRCC.

## Introduction

Clear cell renal cell carcinoma (ccRCC) is the most common malignant subtype of kidney cancer, accounting for 90% of kidney cancers, and demonstrates a high degree of heterogeneity and immunogenicity [1–3]. In recent decades, ccRCC patients have been mainly treated by surgery, and more than 30–50% of ccRCC patients miss the best opportunity for surgery because once metastasis occurs, the 5-year survival rate drops to less than 20% [4, 5]. With the emergence of targeted therapy and immunotherapy, the clinical outcomes of some patients have improved. Although great progress has been made in developing targeted molecular therapies for ccRCC, the therapeutic effect of these strategies is not satisfactory [6]. According to genomic research studies, there is notable molecular and cellular heterogeneity among patients with ccRCC, and differences in gene function are closely related to the progression of ccRCC [7]. The study of the molecular mechanisms underlying ccRCC tumorigenesis and development is helpful to determine new therapeutic targets for ccRCC. However, these molecular mechanisms have not been fully elucidated.

PLAUR (the plasminogen activator urokinase receptor) encodes the receptor of the urokinase plasminogen activator, which may affect many normal and pathological processes related to cell-surface plasminogen activation and local degradation of the extracellular matrix [8]. The activation of plasminogen and extracellular matrix degradation mediated by PLAUR are important causes of tumor metastasis [9]. Overexpression of PLAUR has been observed in many cancers and is usually associated with poor survival and prognosis [10–12]. The abnormal expression of PLAUR is related to the metastasis, invasion, angiogenesis and growth of various cancers, such as pancreatic cancer [13], breast cancer [14] and glioma [15]. In addition, there is evidence that the expression of PLAUR is upregulated in fentinib-resistant lung adenocarcinoma cells, and PLAUR can induce gefitinib resistance in gefitinib-resistant human lung adenocarcinoma cells through the EGFR/p-AKT/survivin signaling pathway [16]. Studies have also shown that abnormal expression of PLAUR in tumors could cause an increase in tumor macrophage infiltration, and these results suggest that PLAUR function may be related to tumor immunity [17]. In addition, some studies have used PLAUR as a tumor immune-related gene to construct prognostic models [18–20]. In addition, Shen et al. constructed a ccRCC immune-related prediction model with PLAUR as one of the many immune-related genes included in the analysis [21]. However, the specific role and mechanism of this gene in ccRCC has not been clearly described in the reported studies, which prompts us to further explore its role and mechanism in ccRCC.

In this study, we comprehensively analyzed the expression and prognostic potential of PLAUR in human tumors using 33 tumor transcriptome datasets from The Cancer Genome Atlas (TCGA) and assessed its role and mechanism in ccRCC with specific focus on its utility in the diagnosis and prognosis of ccRCC. In addition, we analyzed PLAUR clinical, molecular and immunological features in ccRCC to provide new potential insights into the treatment of ccRCC.

## Materials and methods

### Pan-cancer analysis of PLAUR expression

To fully understand the expression of the PLAUR gene in common human cancers, we downloaded the expression profile data of 33 different cancers and the clinical prognosis data from the UCSC database (https://xenabrowser.net/datapages/). We extracted the expression data of the PLAUR gene from the expression profiles of these cancers. At the same time, we analyzed the correlation between the expression of PLAUR in the 33 kinds of cancer and patient prognosis.

### Analysis of PLAUR expression in ccRCC

We downloaded ccRCC expression profile data, methylation data and patients clinical information from TCGA database (https://portal.gdc.cancer.gov/). The PLAUR expression data in ccRCC were extracted, differential expression analysis and visualization were carried out with the R language “limma” package, and diagnostic analysis was carried out with the “pROC” package. Then, the gene expression data were combined with the corresponding patient prognosis data, and metrics of prognosis were analyzed, including disease-specific survival (DSS), progression-free interval (PFI) and overall survival (OS).

Similarly, we extracted PLAUR methylation data from the ccRCC methylation data and analyzed the correlation between gene expression and level of methylation. At the same time, we combined PLAUR methylation data with prognostic data and then analyzed the correlation of gene methylation with patient prognosis.

To understand the correlation between PLAUR expression and the progression of ccRCC, we analyzed the relationship between PLAUR expression and the grade, stage and T stage of ccRCC. In addition, to understand the correlation between PLAUR expression and tumor chemosensitivity, we used the “pRRophetic” package. The “pRRophetic” package is a statistical model created based on the gene expression and drug sensitivity data of a large number of cancer cell lines. It can be used with microarray data of tumor gene expression to predict clinical chemotherapy response [22]. The drugs analyzed in this study included cisplatin, gemcitabine, paclitaxel, sorafenib, sunitinib and pazopanib, all of which are commonly used chemotherapy drugs.

### Prognostic analysis of PLAUR expression in ccRCC

To understand whether the effect of PLAUR expression on the prognosis of ccRCC patients is independent of other clinical factors (sex, age, grade, stage and T stage), univariate Cox and multivariate Cox regression analyses were performed with the R language “survival” package. The “TimeROC” package was used to generate a time-dependent ROC curve.

### Verification of PLAUR expression and analysis of its functional mechanism

We downloaded ccRCC expression profile data from the ICGC (International Cancer Genome Consortium) database and the GSE53757 expression profile dataset and clinical information from the GEO (Gene Expression Omnibus) database. The GSE53757 dataset contained a normal cohort (n = 72) and a ccRCC cohort (n = 72). The expression of PLAUR in these two different cohorts was analyzed using the same analysis method as previously described.

To understand the function and mechanism of PLAUR in ccRCC, we screened PLAUR coexpressed genes based on TCGA expression data. The screening condition was correlations with | r |> 0.4, *P* < 0.01. The DAVID database (https://david.ncifcrf.gov/) was used to perform GO (Gene Ontology) and KEGG (Kyoto Encyclopedia of Genes and Genomes) analyses, and both were screened for differential results with FDR < 0.05. We also used the GSEA method to analyze the pathways affected by PLAUR expression. GSEA 4.1.0 software was used, and the results were considered significant at FDR<0.05. All the above results were visualized using R language software 3.6.1.

### Correlation between PLAUR expression and ccRCC immune infiltration

Based on the expression matrix of ccRCC in TCGA, we calculated the ImmuneScore of ccRCC using the R language “estimate” package, merged the ImmuneScore with the clinical data of patients, and analyzed the correlation between the ImmuneScore and clinical characteristics using the “limma” package. We also calculated the percentage of 21 immune cell types for each sample using the “CIBERSORT” algorithm [23]. *P*<0.05 was used as a filtering condition to remove inaccurate results. The difference in immune cell infiltration content between the high and low PLAUR expression groups was calculated using the wilcox test function and expression was considered to be different at *P*<0.05. In addition, we analyzed the correlation between PLAUR expression and the expression of 10 immune checkpoint genes [24]. The above results are visualized using the “ggpubr” and “vioplot” packages.

## Results

### Pan-cancer analysis of PLAUR expression

**Fig 1:**
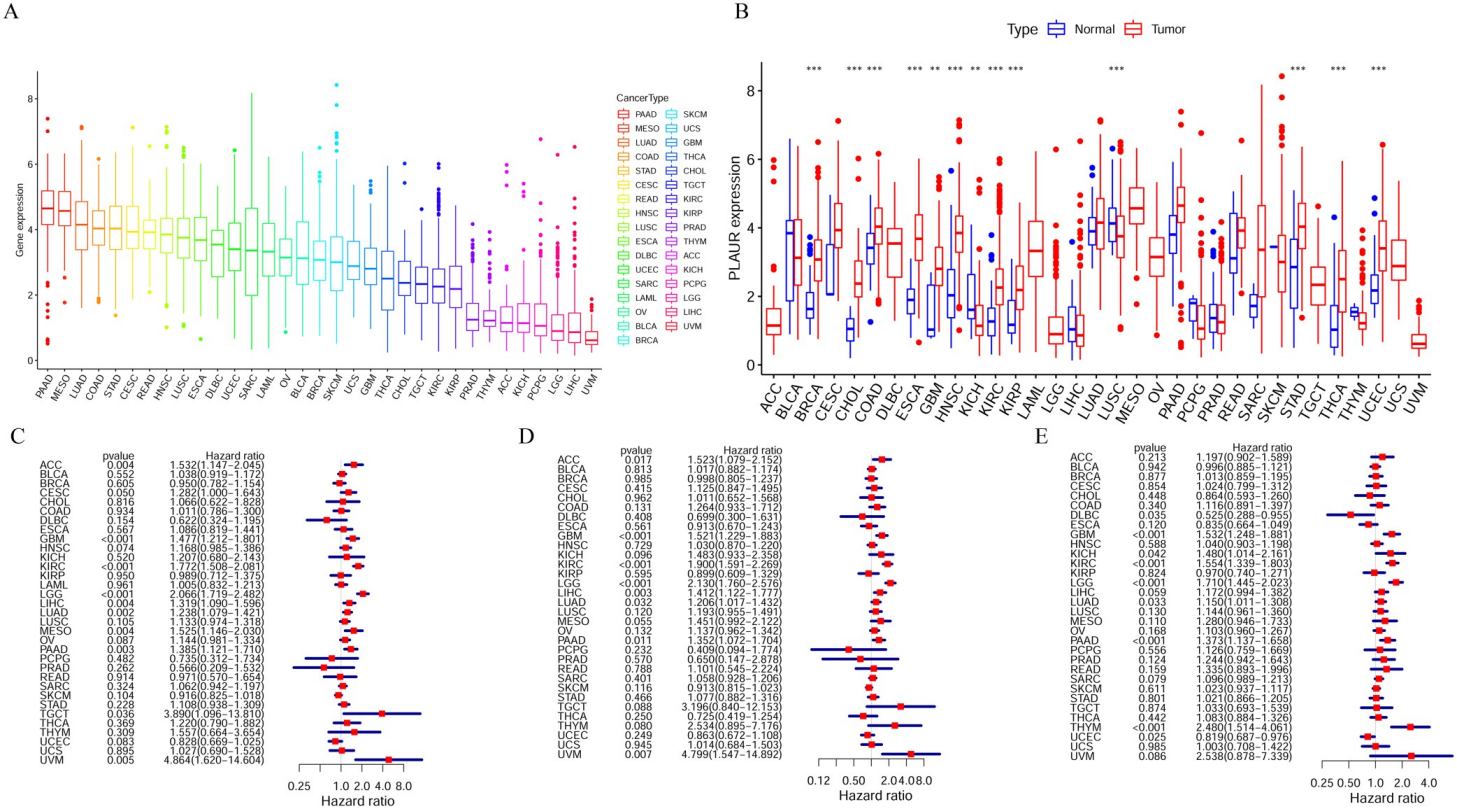
Pan-cancer expression analysis of PLAUR in 33 tumors. A. Ranking of PLAUR expression mean values in 33 tumors; B. Analysis of PLAUR expression in 33 tumors (**P < 0.01, ***P < 0.001); C. Analysis of overall survival (OS) of PLAUR in 33 tumors; D, Analysis of disease-specific survival (DSS) of PLAUR in 33 tumors; E, Analysis of PLAUR in progression-free interval (PFI) analysis in 33 tumors.

Pan-cancer analysis of PLAUR expression helped us comprehensively understand the overall expression of the gene in human tumors. Fig 1A shows the ranking of the mean expression of this gene in 33 tumors, and Fig 1B shows the differential expression of this gene in 33 tumors. The results showed that PLAUR genes were differentially expressed at high levels in BRCA, CHOL, COAD, ESCA, GBM, HNSC, KIRC, KIRP, STAD, THCA and UCEC, while they were differentially expressed at low levels in KICH and LUSC (*P*<0.01). The results of OS analysis, DSS analysis and PFI analysis of PLAUR expression across 33 types of tumors showed that high expression of PLAUR in tumors was significantly correlated with poor prognosis, including in the tumor KIRC (Fig 1C–1E).

### Expression and methylation analysis of PLAUR in ccRCC

**Fig 2:**
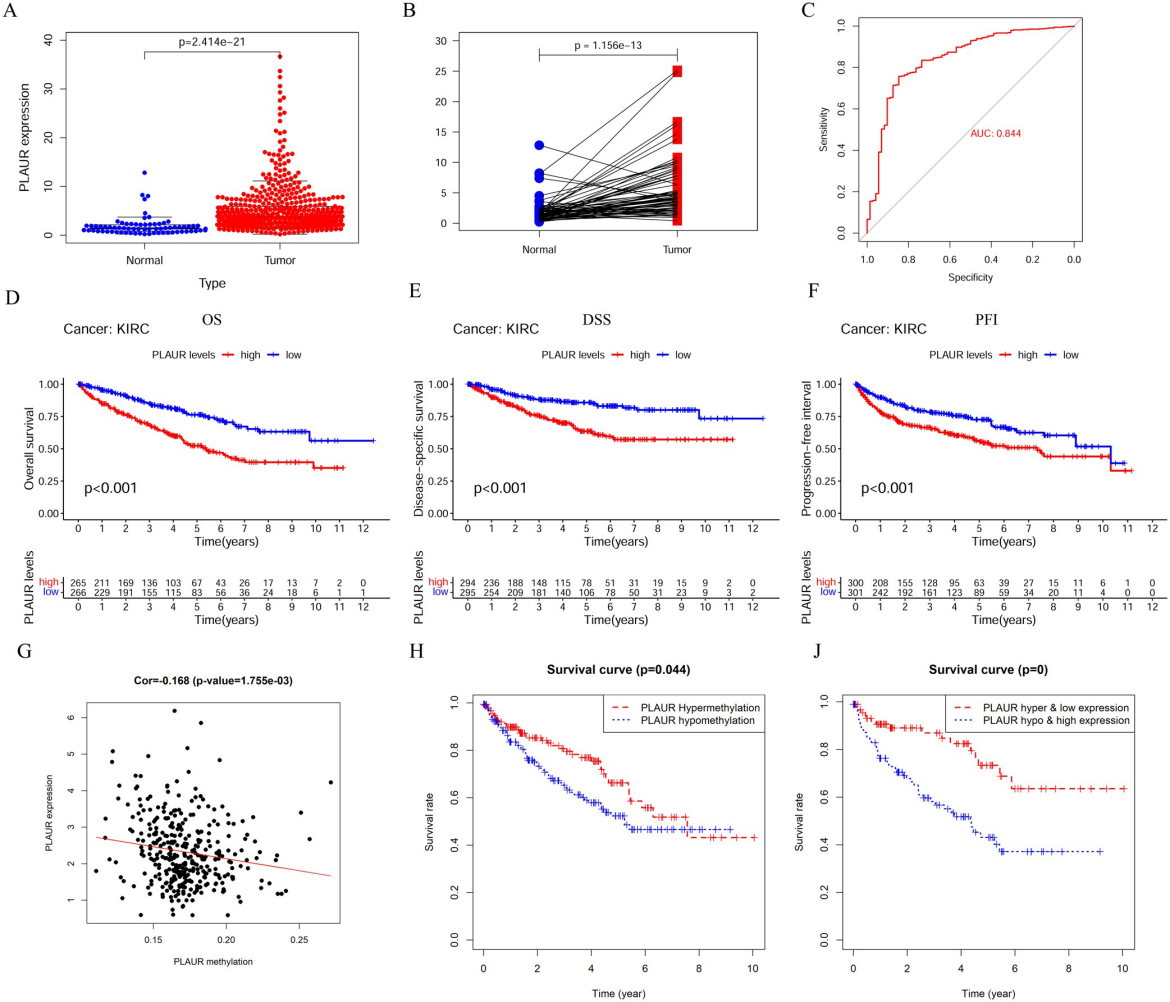
Expression of PLAUR in ccRCC and methylation study. A-F. Analysis of PLAUR expression and prognosis in ccRCC; G-J. Analysis of methylation levels and prognostic correlation of PLAUR in ccRCC.

We extracted the PLAUR expression data in the matrix of ccRCC data for differential expression analysis, and the results showed that PLAUR was significantly highly expressed in ccRCC (Fig 2A). The same results were obtained from the paired analysis of normal and tumor tissues (Fig 2B). To understand whether PLAUR expression might be a diagnostic marker for ccRCC, we performed ROC (receiver operating characteristic curve) analysis, and the results showed that this gene has good diagnostic potential for ccRCC with an area under the curve (AUC) of 0.844 (Fig 2C). The OS, DSS and PFI analysis results showed that high expression of the gene was significantly correlated with poor prognosis (Fig 2D–2F). We also analyzed the methylation of PLAUR in ccRCC, and the degree of methylation of this gene was significantly negatively correlated with its expression (Fig 2G). The results of methylation analysis showed that patients with PLAUR hypermethylation had a better prognosis, which was consistent with the results of expression suppression due to PLAUR hypermethylation (Fig 2H–2J).

**Fig 3:**
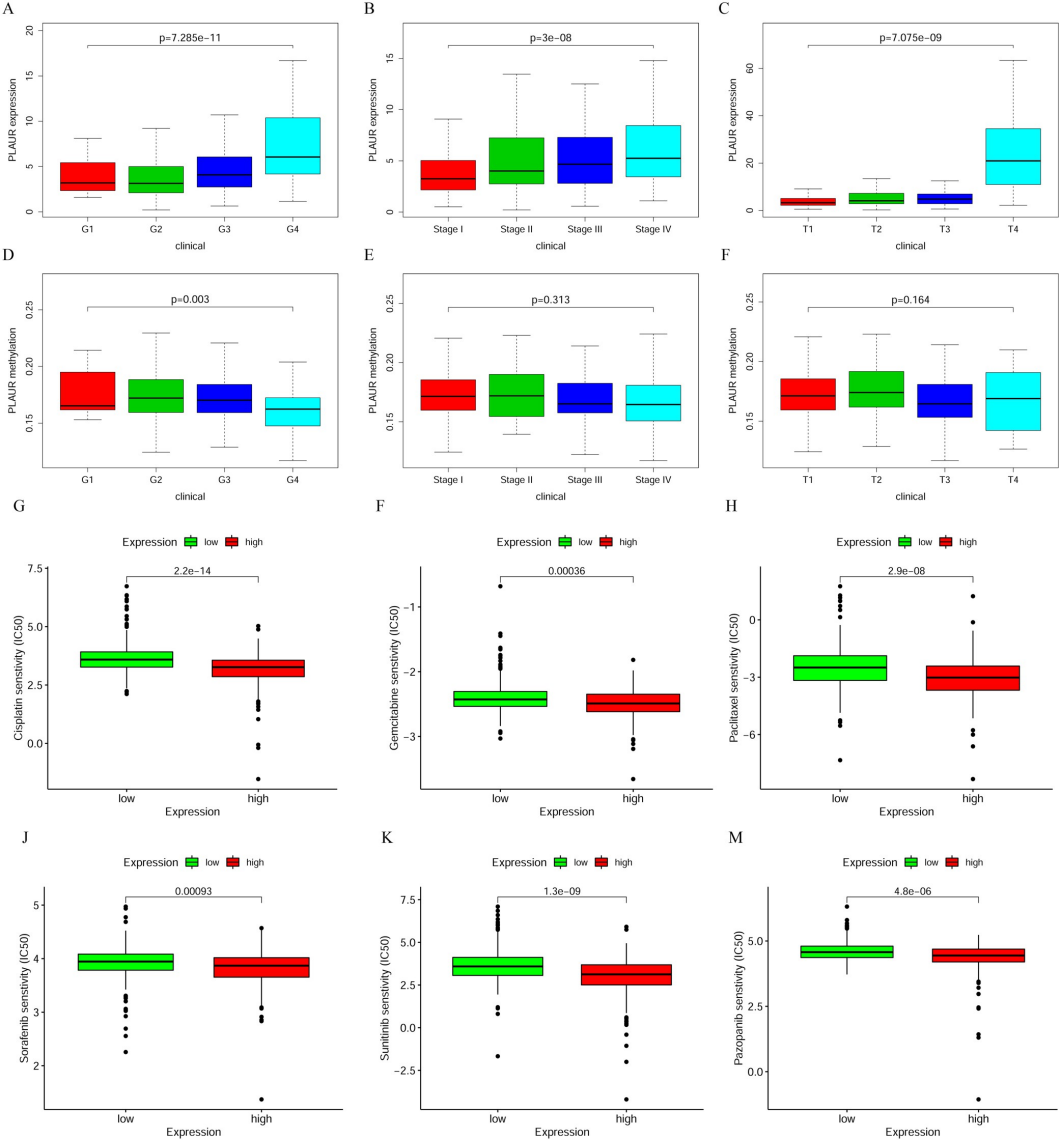
Analysis of the relationship between the expression of PLAUR and the clinical characteristics of patients. Analysis of the correlation between PLAUR expression and ccRCC progression (A-F) and its correlation with chemosensitivity (G-M).

We also analyzed the correlation between PLAUR expression and the clinical features of ccRCC, and our analysis showed that PLAUR expression was significantly related to the grade, stage and T stage of ccRCC and that high PLAUR expression promoted the development of ccRCC (Fig 3A–3F). In addition, we analyzed the correlation between PLAUR methylation and clinical features of ccRCC, and the results showed that hypermethylation of this gene was negatively correlated with grade, stage and T stage. Although the results of the correlation analysis of PLAUR methylation and stage and T stage were not significant, a trend of negative correlation between PLAUR methylation with tumor progression was also observed (Fig 3D–3F). The results of the analysis of correlation between PLAUR expression and sensitivity to commonly used chemotherapeutic agents (cisplatin, gemcitabine, paclitaxel, sorafenib, sunitinib and pazopanib) showed that high PLAUR expression increased tumor sensitivity (Fig 3G–3M; *P* < 0.05).

### Prognostic analysis of PLAUR expression in ccRCC

**Fig 4:**
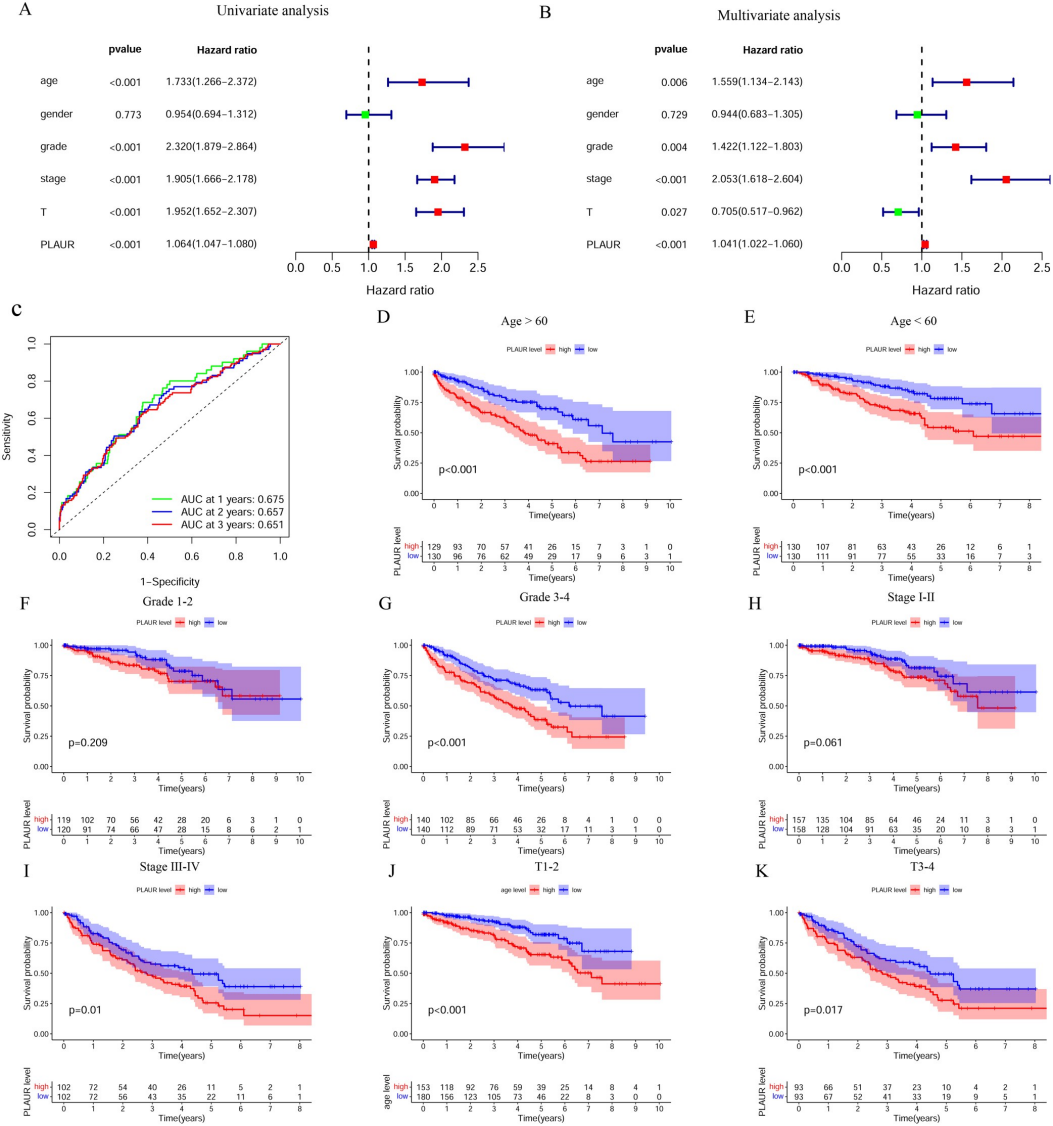
Univariate and multivariate Cox regression analysis and stratified analysis of PLAUR in ccRCC. A. Univariate Cox regression analysis; B. Multivariate Cox regression analysis; C. Prognostic efficacy of PLAUR for ccRCC. D-K. Stratified analysis.

We conducted a univariate Cox regression analysis to further understand whether PLAUR expression can be used as an independent prognostic factor. The results showed that PLAUR expression, age, grade, stage and T stage were all correlated with patient prognosis (Fig 4A). We subsequently performed a multifactorial Cox regression analysis and obtained similar results (Fig 4B), indicating that the expression of this gene was not the only prognostic factor. The results of the time-dependent ROC curve analysis revealed that PLAUR had a good prognostic potential (Fig 4C). In addition, we also conducted a stratified analysis of individual clinical factors and found that high PLAUR expression was significantly correlated with poor prognosis in patients aged > 60 and < 60 years (Figs 3E and 4D). There was no significant correlation between the high expression of PLAUR and the prognosis in the Grade 1–2 group, but there was a significant correlation between the high expression of PLAUR and the poor prognosis in the Grade 3–4 group (Fig 4F and 4G). The prognosis of patients with high expression of PLAUR and stage III-IV tumors was poor (Fig 4H and 4I). High PLAUR expression was significantly associated with poor prognosis in both the T1-2 and T2-4 groups (Fig 4J and 4K). PLAUR expression in the T1-2 group and the T2-4 group was significantly correlated with poor prognosis (Fig 4J and 4K). The results of stratified analysis showed that the expression of PLAUR could predict the prognosis of patients with different clinical features, especially for high-grade ccRCC.

### PLAUR expression validation and analysis of functional mechanisms

**Fig 5:**
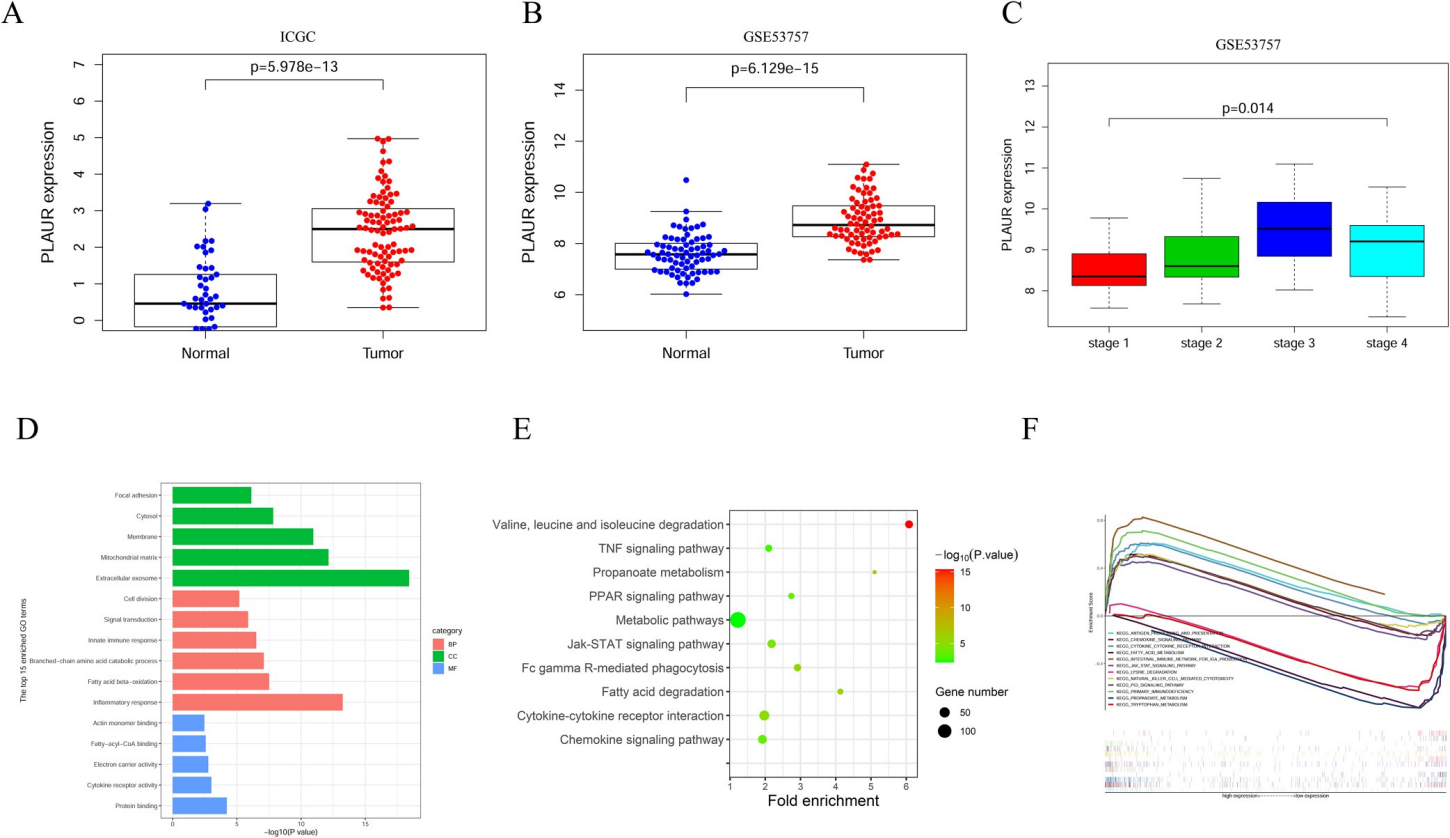
PLAUR expression validation and functional mechanism analysis. A-C. Expression validation analysis of PLAUR in ccRCC; D-E. GO and KEGG enrichment analysis of PLAUR coexpressed genes; F. GSEA pathway enrichment analysis between patients with high and low PLAUR expression.

We performed PLAUR expression analysis with the ccRCC expression profile data obtained from the ICGC database and the GSE53757 dataset obtained from the GEO database. PLAUR was differentially expressed at high levels in ccRCC (Fig 5A and 5B). We also obtained tumor staging data from GSE53757 and found that PLAUR expression was positively correlated with ccRCC progression (Fig 5C). These results are consistent with the analysis results obtained using TCGA.

To understand the role and mechanism of PLAUR action in ccRCC, we obtained data on PLAUR coexpressed genes from TCGA. Pearson correlation coefficients | r | > 0.4 and *P* < 0.01 were used to screen coexpressed genes. A total of 1573 genes were found, including 621 negatively correlated genes and 952 positively correlated genes. The top 20 positively correlated genes and top 20 negatively correlated genes were selected to draw an expression heatmap (S1 Fig). These genes were analyzed by GO and KEGG analyses through the DAVID database. The mechanism of PLAUR function in ccRCC was preliminarily predicted by using *P* < 0.05 as the screening condition. The GO enrichment results of the selected top 15 genes are visualized as shown in Fig 5D and Table 1. These coexpressed genes were mainly enriched in protein binding (BP), inflammatory response (MF) and extracellular exosome (CC) functions. The KEGG enrichment results contained some important pathways, such as the cytokine–cytokine receptor interaction, Jak-STAT signaling pathway, PPAR signaling pathway, TNF signaling pathway and chemokine signaling pathway, which collectively were mainly inflammatory response- and immune-related pathways (Fig 5E; Table 2).

**Table 1:** Results of GO analysis of PLAUR coexpressed genes.

Category	ID	Term	Count	*P* Value	FDR
**MF**	GO:0005515	Protein binding	773	4.45E-08	6.10E-05
**MF**	GO:0004896	Cytokine receptor activity	14	1.42E-06	9.73E-04
**MF**	GO:0009055	Electron carrier activity	22	3.69E-06	0.001685
**MF**	GO:0000062	Fatty-acyl-CoA binding	12	7.80E-06	0.002671
**MF**	GO:0003785	Actin monomer binding	11	1.24E-05	0.003396
**BP**	GO:0006954	Inflammatory response	82	1.38E-17	5.76E-14
**BP**	GO:0006635	Fatty acid beta-oxidation	21	1.47E-11	3.06E-08
**BP**	GO:0009083	Branched-chain amino acid catabolic process	14	5.80E-11	8.07E-08
**BP**	GO:0045087	Innate immune response	73	2.95E-10	3.08E-07
**BP**	GO:0007165	Signal transduction	147	1.61E-09	1.34E-06
**BP**	GO:0051301	Cell division	60	9.37E-09	6.52E-06
**CC**	GO:0070062	Extracellular exosome	342	6.39E-22	3.92E-19
**CC**	GO:0005759	Mitochondrial matrix	70	2.38E-15	7.32E-13
**CC**	GO:0016020	Membrane	258	5.49E-14	1.12E-11
**CC**	GO:0005829	Cytosol	341	9.69E-11	1.49E-08
**CC**	GO:0005925	Focal adhesion	64	6.06E-09	7.44E-07

**Table 2:** Results of KEGG analysis of PLAUR coexpressed genes.

ID	Term	Count	*P* value	FDR
**hsa00280**	Valine, leucine and isoleucine degradation	28	4.61E-16	1.11E-13
**hsa00640**	Propanoate metabolism	14	6.26E-07	1.88E-05
**hsa00071**	Fatty acid degradation	17	8.63E-07	2.07E-05
**hsa04060**	Cytokine–cytokine receptor interaction	47	7.02E-06	1.40E-04
**hsa04630**	Jak-STAT signaling pathway	31	5.19E-05	8.89E-04
**hsa03320**	PPAR signaling pathway	18	1.75E-04	0.002213
**hsa04062**	Chemokine signaling pathway	35	2.25E-04	0.002698
**hsa04668**	TNF signaling pathway	22	0.001382	0.011841
**hsa01100**	Metabolic pathways	146	0.004246	0.02754
**hsa04666**	Fc gamma R-mediated phagocytosis	24	3.28E-06	7.16E-05

To ensure the accuracy of the pathway prediction results, we used the GSEA method to predict the pathways that may be affected by PLAUR. We identified several inflammatory responses and immune-related pathways in our results, such as primary immunodeficiency, chemokine signaling pathway, cytokine—cytokine receptor interaction, intestinal immune network for IgA production and natural killer cell mediated cytotoxicity. These results suggest that PLAUR may be involved in the regulation of the immune microenvironment in ccRCC (Fig 5F; Table 3).

**Table 3:** Analysis of PLAUR functions by the GSEA method.

Pathways	ES	NES	NOM p-val	FDR q-val
**fatty acid metabolism**	-0.78821	-2.18234	0	0.042222
**lysine degradation**	-0.61576	-1.99962	0.012048	0.04971
**propanoate metabolism**	-0.79639	-2.12337	0	0.027284
**tryptophan metabolism**	-0.65001	-2.09008	0	0.032831
**antigen processing and presentation**	0.608715	2.006461	0.018072	0.023897
**primary immunodeficiency**	0.714291	1.902942	0.022774	0.033832
**chemokine signaling pathway**	0.516997	1.905894	0.011976	0.035143
**cytokine cytokine receptor interaction**	0.609397	2.519677	0	0
**intestinal immune network for iga production**	0.826802	2.429305	0	2.60E-04
**jak stat signaling pathway**	0.453581	1.87288	0.01518	0.035929
**natural killer cell mediated cytotoxicity**	0.514903	1.913119	0.013672	0.035358
**p53 signaling pathway**	0.499782	1.816742	0.018975	0.046898

Abbreviations: ES, enrichment score; FDR, false discovery rate; GSEA, gene set enrichment analysis; NES, normalized enrichment score; NOM, nominal.

### Correlation analysis of PLAUR expression and degree of immune cell infiltration

**Fig 6:**
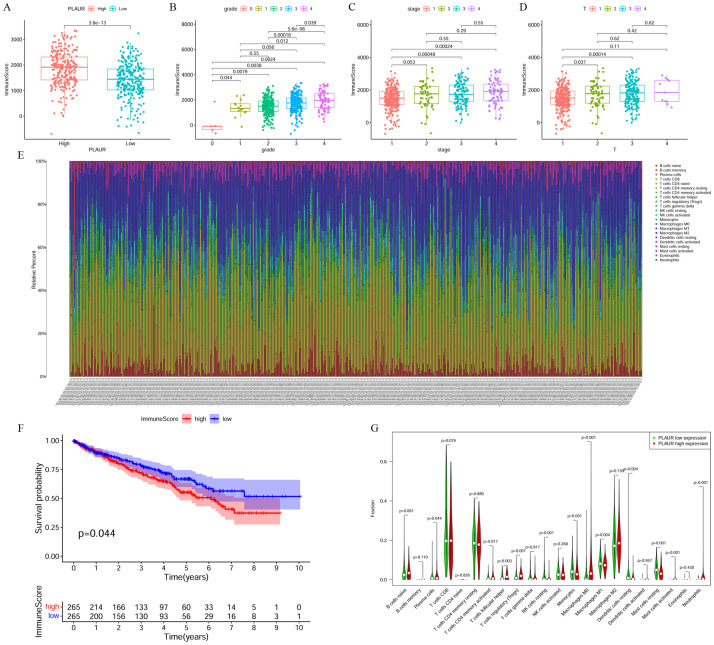
Correlation analysis between PLAUR expression and immune cell infiltration. A. Correlation between high and low PLAUR expression and ImmuneScore; B. Correlation between Grade and ImmuneScore; C. Correlation between Stage and ImmuneScore; D. Correlation between T stage and ImmuneScore; E. Immune cell infiltration level stacking graph; F. Correlation between ImmuneScore and ccRCC patient prognosis correlation analysis; G. Immune cell infiltration level and PLAUR expression correlation analysis.

**Fig 7:**
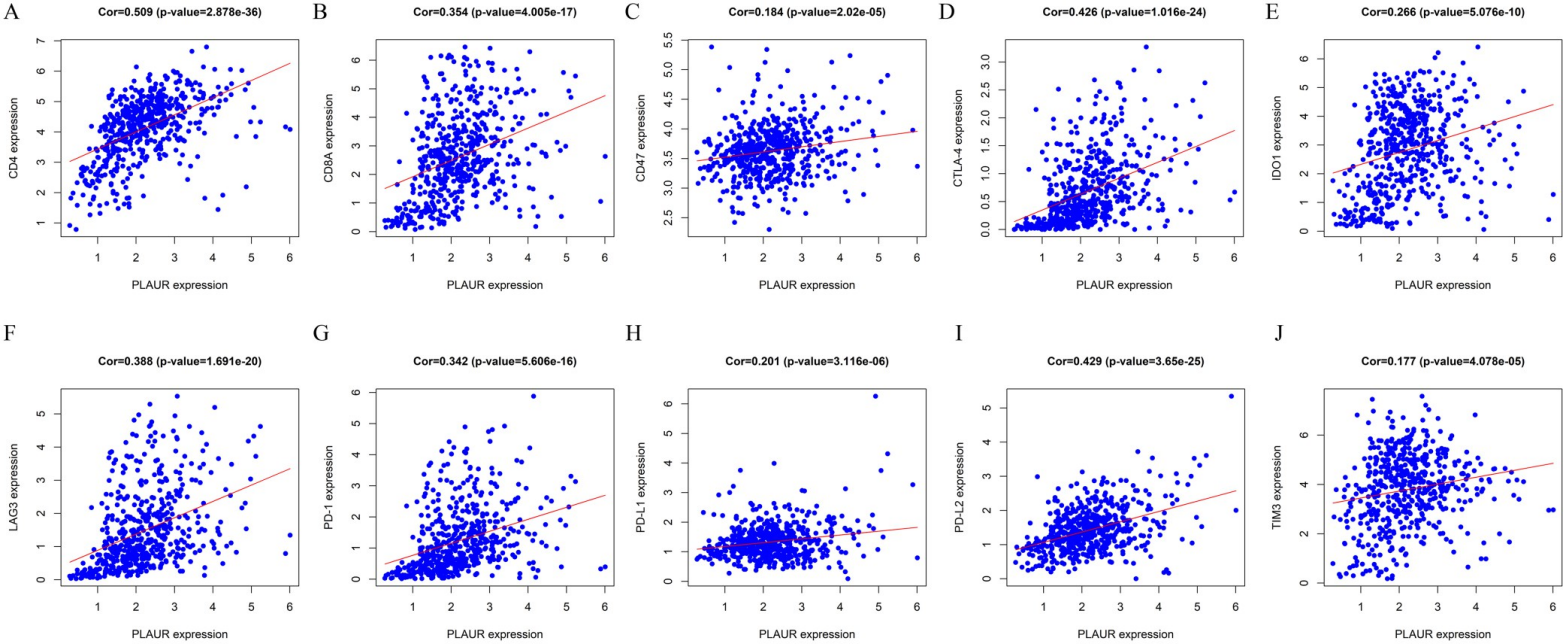
Correlation analysis of PLAUR expression and immune checkpoint gene expression.

To investigate whether PLAUR expression affects the degree of immune cell infiltration of ccRCC, we calculated the ImmuneScore for each ccRCC sample and then analyzed the correlation between the ImmuneScore and the clinical information of ccRCC patients. We found that the ImmuneScore was significantly higher in the high PLAUR expression samples than in the low PLAUR expression samples (Fig 6A). Moreover, the ImmuneScore was significantly higher in the high-grade and high-stage groups (Fig 6B–6D) than in the low-grade and low-stage groups, and prognostic correlation analysis also found that patients with a high ImmuneScore had a poorer prognosis (Fig 6F). We then used the "CIBERSORT" algorithm to calculate the relative degree of immune cell infiltration in each sample and drew an immune cell infiltration abundance map to show the results of each type of immune cell infiltration in each sample (Fig 6E). We found that PLAUR expression was significantly correlated with the infiltration of some immune cells (Fig 6G; Table 4). We also analyzed the correlation of PLAUR expression with the expression of the immune checkpoint genes PD-1, PD-L1, PD-L2, CTLA-4, LAG-3, CD47, CD4, CD8A, IDO1 and TIM-3 and found that PLAUR expression was significantly positively correlated with that of all of these genes (Fig 7). These results suggest that PLAUR expression is associated with the degree of immune cell infiltration and that PLAUR may become a target for immunotherapy.

**Table 4:** Correlation analysis between PLAUR expression and the relative content of immune cell infiltration.

Cell type	Correlation	*P* value
**B cells naïve**	0.074436	0.084254
**B cells memory**	0.000216	0.995999
**Plasma cells**	0.160366	0.000185
**T cells CD8**	0.007185	0.867824
**T cells CD4 memory resting**	-0.08805	0.041
**T cells CD4 memory activated**	0.199827	2.93E-06
**T cells follicular helper**	0.144968	0.000736
**T cells regulatory (Tregs)**	0.267829	2.63E-10
**T cells gamma delta**	0.043241	0.316331
**NK cells resting**	-0.19006	8.88E-06
**NK cells activated**	-0.13264	0.002029
**Monocytes**	-0.16026	0.000187
**Macrophages M0**	0.366262	1.48E-18
**Macrophages M1**	-0.12409	0.003908
**Macrophages M2**	0.029855	0.489028
**Dendritic cells resting**	-0.11554	0.007249
**Dendritic cells activated**	-0.0551	0.20154
**Mast cells resting**	-0.41204	1.67E-23
**Mast cells activated**	0.1908	8.18E-06
**Eosinophils**	-0.09803	0.022843
**Neutrophils**	0.161798	0.000162

## Discussion

Patients with advanced renal clear cell carcinoma have a high mortality rate, and treating metastatic renal cell carcinoma remains a huge challenge [25]. At present, traditional clinical treatments, such as surgical resection, radiotherapy and chemotherapy, are not effective for this highly invasive tumor [26]. However, some studies have reported that targeted therapies for ccRCC could be better treatments; for example, VHL is considered a potential molecular target for ccRCC, and targeting the VHL/VEGFR pathway may play an important role in the treatment of advanced ccRCC [27]. Therefore, identifying and predicting new biological targets is essential for developing new drugs and prolonging the survival of ccRCC patients.

Pan-cancer bioinformatic analysis has been gradually developed along with the recent advancements in the tumoromics database. Pan-cancer analysis can easily perform panoramic analysis of many common human tumors at the same time, which greatly helps researchers quickly understand the value of the studied molecular target and has been applied in many tumor-related studies [28–30]. In our research, we analyzed the relationship between the expression of PLAUR and prognosis in 33 common human tumors and systematically evaluated the importance of PLAUR in human tumors. Then, we shifted the research focus of this gene to ccRCC, which we are most interested in. We further studied the clinical and molecular characteristics of our target gene in ccRCC and evaluated its potential role in the development and progression of ccRCC. Studies have shown that PLAUR is highly expressed in many tumors, including ccRCC [21, 31]. This is consistent with our results. However, these studies only focus on the relationship between the expression of PLAUR and prognosis, and there is no in-depth study on the role and mechanism of this gene in ccRCC. In addition, our study also found that there was a significant correlation between high PLAUR expression and tumor stage and grade. The degree of methylation of the gene in ccRCC is also related to tumor progression, and the prognosis is better in patients with the low PLAUR expression caused by hypermethylation. We also found that the expression of this gene has good diagnostic potential (AUC = 0.844), which is helpful for the discovery of new diagnostic markers of ccRCC. We also analyzed the relationship between PLAUR expression and the sensitivity of ccRCC to tumor chemotherapeutic drugs, and the results suggest that the expression of this gene is correlated with ccRCC sensitivity to many common chemotherapeutic drugs, and thus targeting this gene therapeutically may help to overcome the problem of chemotherapy resistance in ccRCC.

To understand the mechanism of PLAUR action in ccRCC, we used the DAVID database for enrichment analysis of the coexpressed genes of PLAUR. We also analyzed the possible pathways involved in PLAUR function by GSEA, and the results suggested that the gene was involved in the inflammatory and immune-related pathways of ccRCC. This is consistent with the conclusion that PLAUR can be used in predictive models as one of the genes related to immune regulation [18]. The study of the tumor immune microenvironment has gradually become a focus in the field of oncology research, and previous studies have paid much attention to the role of genes in the immune microenvironment. Previous studies have focused more on the role of PLAUR expression in tumors and patient prognosis, and very few studies have focused on the role of this gene in the immune microenvironment of tumors [12, 32, 33]. The role of PLAUR in the immune microenvironment in ccRCC has not been reported. Therefore, this prompted us to further study the correlation between PLAUR expression and degree of immune cell infiltration in ccRCC. In contrast to previous studies, we analyzed the relationship between PLAUR expression and degree of immune cell infiltration in ccRCC based on the "CIBERSORT" algorithm, and our results indicated that PLAUR expression was positively correlated with the degree of infiltration of immune cells, such as plasma cells, T cells, M0 macrophages and neutrophils. We also found that PLAUR expression negatively correlated with the infiltration of some immune cells, such as resting memory CD4 T cells, NK cells and resting mast cells.

Tumor immunotherapy has become a newly developed therapy in recent years. It is considered to be an effective treatment for cancer [34]. ccRCC is highly immunogenic and is characterized by a large degree of immune cell infiltration. However, cancer cells can evade immune attack by creating an immunosuppressive environment through various mechanisms, such as disruption of effective antigen presentation, downregulation of effector T cell action, upregulation of immune tolerance promotion and activation of T cell "incompetence" pathways, and thus immune evasion is an important cause of tumorigenesis and progression. Therefore, the tumor microenvironment is closely related to the occurrence and development of ccRCC [35]. In recent years, the role of immune checkpoints in tumor therapy has been gaining attention, and tumor immunotherapy is expected to be an excellent treatment to completely kill tumor cells [36–38]. Our results revealed that PLAUR expression is positively correlated with the expression of many well-known immune checkpoint genes, suggesting that the use of immune checkpoint inhibitors along with inhibition of PLAUR expression in ccRCC patients may achieve better therapeutic results. In addition, the expression of PLAUR is related to the degree of infiltration of many immune cells, which is a factor worth considering in the application of immunotherapy. The present study also has some shortcomings, and sufficient samples are needed in the future to verify the role of PLAUR in ccRCC and the mechanisms of PLAUR function related to the regulation of immune infiltration.

In conclusion, we extensively investigated the role of PLAUR by bioinformatics in a variety of tumors, and in particular, we found that PLAUR is highly expressed in human ccRCC tissues. We also found a correlation between PLAUR expression levels and clinical features in ccRCC patients. The mechanism by which PLAUR promotes ccRCC development through the immune microenvironment was preliminarily elucidated in this study, indicating that PLAUR may be a potential therapeutic target.

## Supporting information

S1 Fig Expression heatmap of PLAUR coexpressed genes. (TIF)
