# Supplementary figures and images for: Comprehensive analysis of the importance of PLAUR in the progression and immune microenvironment of renal clear cell carcinoma
Source: PLoS One. 2022 Jun 8;17(6):e0269595. doi: 10.1371/journal.pone.0269595 (PMC9176830; doi:10.1371/journal.pone.0269595)

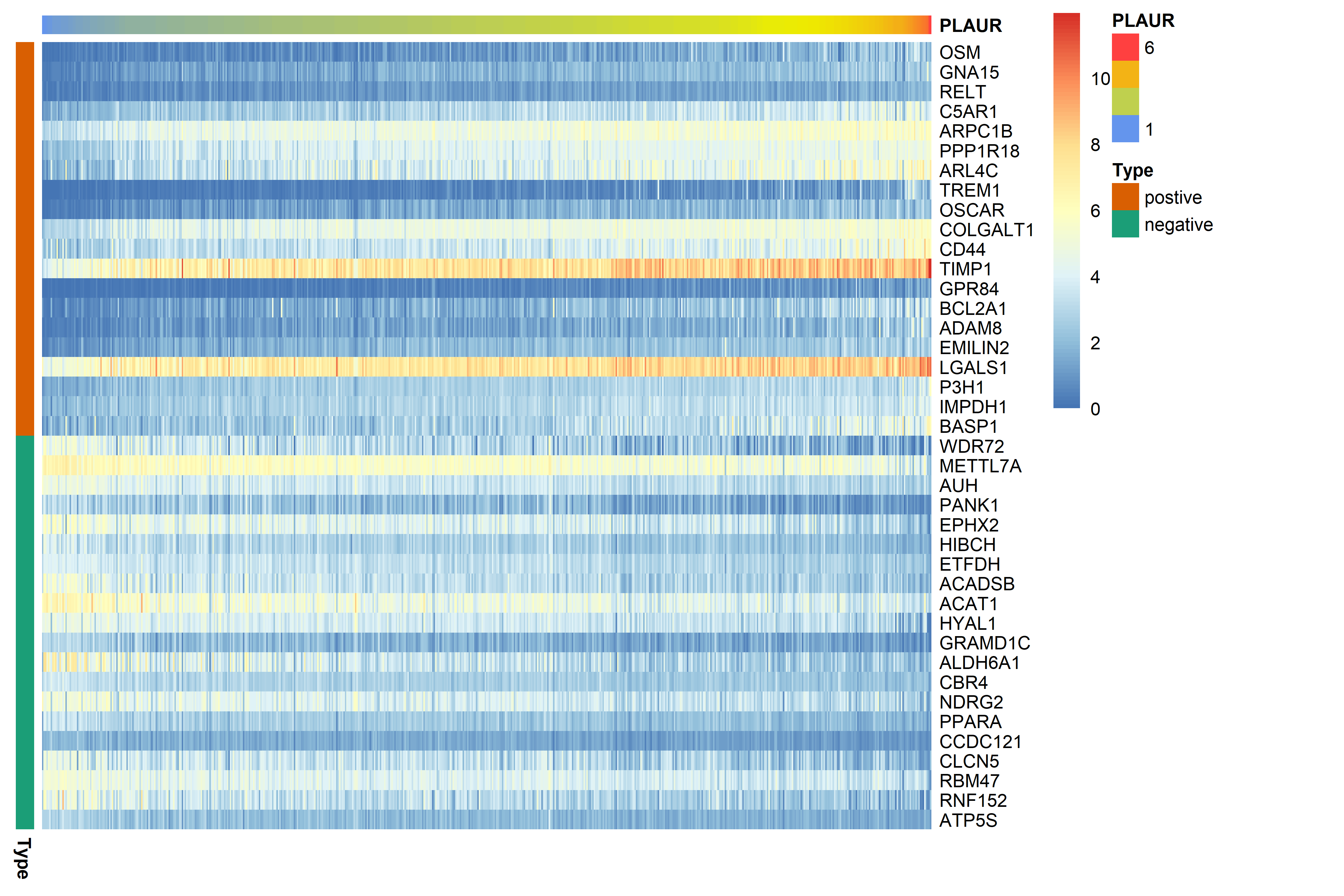

Supplement: S1 Fig — (TIF) [file pone.0269595.s001.tif]
